# Fabrication and Characterization of an Ag–AgPd Thick-Film Thermopile Heat-Flux Sensor for High-Temperature Applications

**DOI:** 10.3390/s26134030

**Published:** 2026-06-25

**Authors:** Zhichun Liu, Fei Chen, Zhixuan Su, Heng Wang, Jinghan Si, Junyang Chen, Zihan Du, Zhenyin Hai

**Affiliations:** School of Aerospace Engineering, Xiamen University, Xiamen 361005, China

**Keywords:** Ag–AgPd, heat flux sensor, thick film thermopile, high-temperature measurement, dynamic response

## Abstract

High-temperature metallic surfaces in aero-engine hot sections and related thermal systems are subjected to MW/m^2^-level heat-flux loads and transient thermal conditions, creating a need for sensors capable of quantifying heat flux under high-temperature conditions. This study aims to develop a screen-printed Ag–AgPd thick-film thermopile heat-flux sensor (HFS) for MW/m^2^-level heat-flux measurement on high-temperature metallic surfaces. Its main feature is the integration of an Ag–AgPd thermopile sensing layer, an insulating layer, and a thermal-resistance layer on a SUS430 stainless-steel substrate through a screen-printing-based multilayer fabrication route. Microstructural characterization, annealing condition comparison, laser comparison calibration, repeated loading, dynamic-response testing, and flame-heating testing were conducted to evaluate the sensor structure and performance. Under laser comparison calibration, the sensor achieved a MW/m^2^-level calibrated heat-flux response over 0.32–1.37 MW/m^2^, with a near-linear output relationship of $R^{2}>0.998$, a sensitivity of 2.67 μV/(kW/m^2^), a nonlinearity of 1.83%, a hysteresis error below 0.29%, a repeatability error below 0.43%, a sample-to-sample consistency error of 1.06%, a maximum accuracy-test deviation of 1.84%, and a maximum repeated-loading stability error of 1.33%. The sensor also exhibited a time constant of 0.806 s under laser step excitation, and the baseline-corrected equivalent heat-flux response remained stable during approximately 120 s of flame heating at about 800 °C. These results indicate that the proposed HFS provides a feasible thick-film thermopile sensing approach for MW/m^2^-level heat-flux measurement on high-temperature metallic surfaces.

## 1. Introduction

Hot-section metallic components in aero-engines, power-generation systems, and other high-temperature thermal equipment are commonly exposed to MW/m^2^-level thermal loads, steep temperature gradients, and transient heating. Combustor liners, nozzles, turbine-related structures, and other metallic surfaces experience unsteady heat-flux fluctuations during operating transitions, and short-duration heat-flux peaks can strongly affect thermal fatigue, structural reliability, cooling design, and thermal-protection evaluation [1,2,3,4]. Unlike temperature, heat flux describes the rate at which thermal energy enters or leaves a solid surface. Therefore, direct heat-flux measurement on metallic surfaces is important for quantifying local heat-transfer behavior and supporting the validation of high-temperature thermal-management designs and numerical simulations [5,6].

Heat flux in high-temperature components is often estimated using indirect methods, including temperature measurement combined with inverse heat-conduction analysis or conjugate heat-transfer modeling [7,8]. These approaches can provide useful boundary information, but their accuracy depends on thermophysical properties, boundary-condition assumptions, spatial discretization, and numerical models, particularly during strongly transient thermal loading. Infrared thermography and endoscopic infrared techniques can obtain non-contact temperature-field information with high spatial resolution [9,10]. However, the measurement result is affected by surface emissivity, viewing angle, surface oxidation, and optical access at elevated temperature. More importantly, these approaches measure temperature or rely on model reconstruction, rather than directly sensing the heat flux passing through the surface.

Thin-film and thick-film HFSs have been investigated for high-temperature and harsh-environment measurements because they can be integrated onto solid surfaces and directly convert thermal loading into electrical signals [11,12,13,14]. Thin-film sensors can offer low heat capacity and compact layouts, whereas thick-film sensors provide several practical advantages for engineering fabrication. In particular, screen printing can be performed under atmospheric conditions, does not require vacuum deposition equipment, and is suitable for forming relatively thick functional layers over a defined area. The process also allows flexible control of film thickness, scalable patterning, and multilayer integration through printing and sintering [13,15]. These features are attractive for metallic-surface heat-flux monitoring, where the sensing unit must maintain electrical insulation, provide sufficient thermal resistance, and survive repeated high-temperature processing.

For a thermopile-type HFS, the applied heat flux induces a temperature difference across the thermal-resistance layer, and the Ag–AgPd thermopile converts the temperature difference into voltage through the Seebeck effect [6,16]. The output voltage is therefore related to both the thermal-resistance structure and the thermoelectric behavior of the paired legs. In this configuration, the design of the multilayer insulation, thermopile, and thermal-resistance layers is central to the static calibration response, repeated-loading behavior, and transient output stability of the sensor.

Ag and AgPd thick films are compatible with screen-printing and high-temperature sintering processes and have been used in printed electronic and high-temperature thick-film structures [17,18]. In this work, Ag and AgPd were selected as the paired thermoelectric legs of the thermopile. The Ag–AgPd material pair provides a thermoelectric output under a temperature difference, while the high-temperature annealing process helps stabilize the microstructure and electrical output of the multilayer sensing unit. Based on this design, an Ag–AgPd thick-film thermopile structure was adopted for heat-flux sensing on SUS430 stainless-steel substrates.

The objective of this work is to develop and evaluate an Ag–AgPd thick-film thermopile HFS for MW/m^2^-level heat-flux measurement on high-temperature metallic surfaces. This work features a screen-printing-based multilayer integration of an Ag–AgPd thermopile sensing layer, an insulating layer, and a thermal-resistance layer on a SUS430 stainless-steel substrate, providing a thick-film route for direct heat-flux sensing on high-temperature metallic surfaces. To achieve this objective, an Ag–AgPd thick-film thermopile HFS was designed and fabricated on a SUS430 stainless-steel substrate by screen printing and multi-step sintering. The fabrication process, multilayer structure, annealing-induced microstructural evolution, static calibration characteristics, repeated-loading behavior, dynamic response, and flame-heating behavior were systematically investigated. From an application perspective, such a sensor can provide direct heat-flux information for online thermal condition monitoring of high-temperature metallic components and may help us identify abnormal thermal-load variations, cooling-performance degradation, or local overheating tendencies in aero-engine hot sections and related thermal systems.

## 2. Materials and Methods

### 2.1. Materials

A silicate-based composite material (07H-1114L, Dongguan Saiqin Electronics Technology Co., Ltd., Dongguan, China) was used to prepare the insulating and thermal-resistance layers. The material was mainly composed of silicon dioxide (${\mathrm{SiO}}_{2}$), aluminum oxide (${\mathrm{Al}}_{2}O_{3}$), and calcium oxide (CaO). Ag paste (Chengdu Hehe Micro-Nano Technology Co., Ltd., Chengdu, China) and AgPd paste (02H-1805, Dongguan Saiqin Electronics Technology Co., Ltd., Dongguan, China) were used as the thermoelectric thick-film materials. The substrate was SUS430 stainless steel (Dongguan Haitong Metal Materials Co., Ltd., Dongguan, China) with dimensions of 30 mm × 20 mm × 1 mm. ${\mathrm{Al}}_{2}O_{3}$ solder pads with a diameter of 3 mm and a thickness of 0.5 mm were used for lead connection. Each solder pad contained a rectangular groove with a depth of 0.2 mm and dimensions of 3 mm × 0.3 mm for embedding a 0.3-mm-diameter Ag wire. A terpineol-based diluent (Dongguan Saiqin Electronics Technology Co., Ltd., Dongguan, China) was used to adjust the viscosities of the Ag and AgPd pastes, and polyimide was used as the mask material.

### 2.2. Design and Fabrication

**Figure 1:**
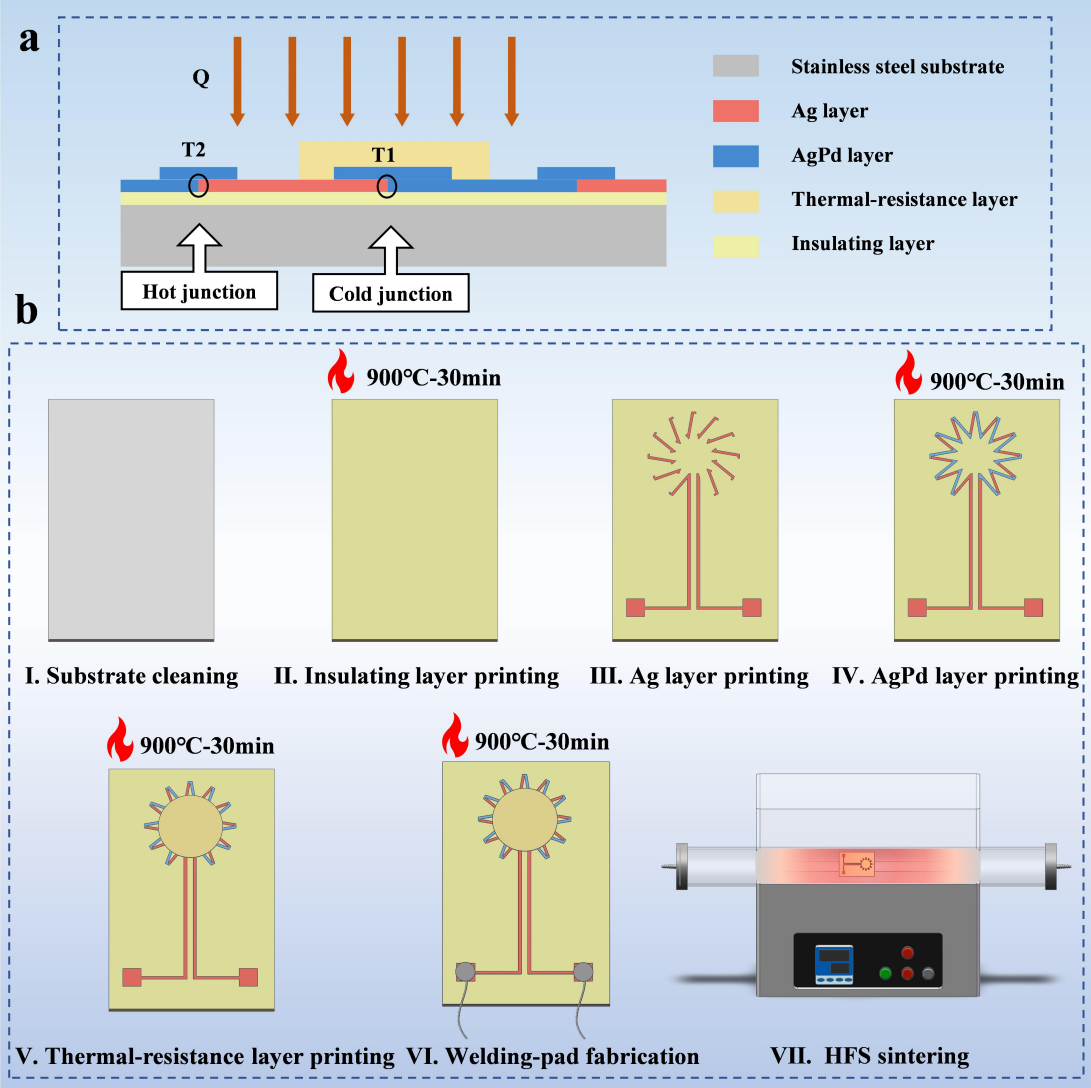
Structure and fabrication process of the Ag–AgPd thick-film thermopile HFS. (**a**) Schematic diagram of the thermopile-type HFS structure and sensing principle. (**b**) Fabrication process of the Ag–AgPd thick-film HFS.

The Ag–AgPd thick-film thermopile HFS was designed on a SUS430 stainless-steel substrate, as shown in Figure 1a. The sensing unit consisted of an insulating layer, an Ag–AgPd thermopile sensing layer formed by 11 pairs of Ag–AgPd thermocouples, a thermal-resistance layer, ${\mathrm{Al}}_{2}O_{3}$ solder pads, and 0.3 mm diameter Ag wires. The thermal-resistance layer provides the through-thickness thermal resistance required to establish the temperature difference across the Ag–AgPd thermopile. The SUS430 stainless-steel substrate provided mechanical support for the sensor structure [19]. The insulating layer electrically isolated the thermopile from the SUS430 stainless steel substrate, while the thermal-resistance layer was printed over the sensing region to establish a temperature difference under through-thickness heat-flux loading. The Ag–AgPd thermopile was arranged to convert this temperature difference into an output voltage through the Seebeck effect.

The detailed theoretical derivation of the sensing principle is provided in Section S1 of the Supplementary Information. According to Equations (S1)–(S4), and as illustrated in Figure 1a, when heat-flux density *q* is applied to the sensor surface, the thermal-resistance layer induces a temperature difference across the thermopile, and the Ag–AgPd thermopile converts this temperature difference into an output voltage.

The fabrication process for the Ag–AgPd thick-film HFS is shown in Figure 1b. The SUS430 stainless-steel substrate was first ultrasonically cleaned in acetone, deionized water, and ethanol for 5 min each. The insulating layer (20 mm × 30 mm) was then screen-printed, dried at 150 °C for 5 min, and sintered at 900 °C for 30 min in air. To improve printability, the Ag paste and AgPd paste were diluted with the terpineol-based diluent at mass ratios of 9:1 and 8:2, respectively. The Ag and AgPd layers with a line width of 0.26 mm were printed using the polyimide mask, dried at 150 °C for 5 min, and sintered at 900 °C for 30 min. The thermal-resistance layer (30 mm × 20 mm) was subsequently printed over the sensitive area, dried at 150 °C for 5 min, and sintered at 900 °C for 30 min. Finally, the ${\mathrm{Al}}_{2}O_{3}$ solder pads and 0.3-mm-diameter Ag wires were connected using AgPd paste, followed by drying and sintering at 900 °C for 30 min to complete the electrical leads.

The thicknesses of the printed layers were characterized by profilometry. The Ag layer, AgPd layer, and insulating layer were approximately 11.07, 9.46, and 31.35 μm thick, respectively, confirming the formation of the multilayer thick-film structure. After completion of the multilayer fabrication process, the Ag–AgPd thick-film samples were subjected to an additional post-annealing treatment at 900 °C for 30, 60, 90, and 120 min to evaluate the effect of annealing duration on the microstructure and output behavior. Except for the post-annealing duration, all fabrication parameters were kept unchanged. The sintering and post-annealing steps were performed in air.

### 2.3. Testing and Characterization

The experimental testing system consisted of a tube furnace, a fiber laser heating source, a calibrated reference heat-flux sensor, a water-cooling unit, a power controller, and a data-acquisition system. The tube furnace (OTF-1200X, Hefei Kejing Material Technology Co., Ltd., Hefei, China) was used for high-temperature sintering and annealing of the thick-film layers. A 1064-nm fiber laser (YLM-QCW, IPG Photonics, Marlborough, MA, USA) with a maximum power of 300 W was used as the controllable heat source for static heat-flux calibration and laser step excitation. The output voltage of the fabricated HFS was recorded using a digital multimeter/data-acquisition unit (DAQ6510, Tektronix, Beaverton, OR, USA). A GD-C0-5M heat-flux sensor (Xi’an Kretek Science and Trade Co., Ltd., Xi’an, China) was used as the calibrated reference sensor, and the water-cooling unit was used to maintain a stable cooling boundary during laser-heating tests. Detailed information on the testing equipment is provided in Section S5 of the Supplementary Information.

Static calibration was performed by a comparison calibration method. The fabricated Ag–AgPd thick-film HFS and the GD-C0-5M reference sensor were tested under nominally identical laser-heating conditions in an equivalent arrangement, so that the output of the fabricated HFS could be correlated with the heat-flux density measured by the calibrated reference sensor. During calibration, the laser power was adjusted stepwise to generate different heat-flux levels, and the cooling-water temperature was maintained at 25 °C. The laser spot diameter on the sensing surface was approximately 10 mm, and the laser-to-sensor distance was 75 cm. The voltage signal was sampled at approximately 40 Hz, and each heat-flux level was held until both the reference heat-flux density and the HFS output reached stable states. Each calibration condition was repeated three times, and the steady-state values were used for calibration. The hysteresis, repeatability, sample-to-sample consistency, accuracy-test deviation, and repeated-loading stability metrics were calculated according to the definitions provided in the Supplementary Information and related heat-flow-meter evaluation methods [20]. Error bars, where shown, represent the dispersion metric defined for the corresponding dataset in the Supplementary Information.

To evaluate the dynamic response of the fabricated HFS, a laser step-heating test was performed by applying a sudden thermal excitation to the sensing region. The transient voltage response was recorded by the data acquisition system, and the time constant was defined as the time required for the output voltage to reach 63.2% of its steady-state value. To examine the output stability under direct high-temperature exposure, a flame-heating test was conducted using a flame torch. A K-type thermocouple was used to monitor the local temperature near the sensing region, and the output voltage of the HFS was recorded simultaneously.

## 3. Results and Discussion

### 3.1. Microstructure and Annealing-Time Effect

Because sintering and annealing conditions can influence the microstructure and output stability of thick-film materials [21], the effect of annealing time on the Ag–AgPd thick-film structure was investigated as a fabrication-parameter comparison. The samples were annealed at 900 °C for 30, 60, 90, and 120 min, respectively, while the other fabrication parameters were kept unchanged.

**Figure 2:**
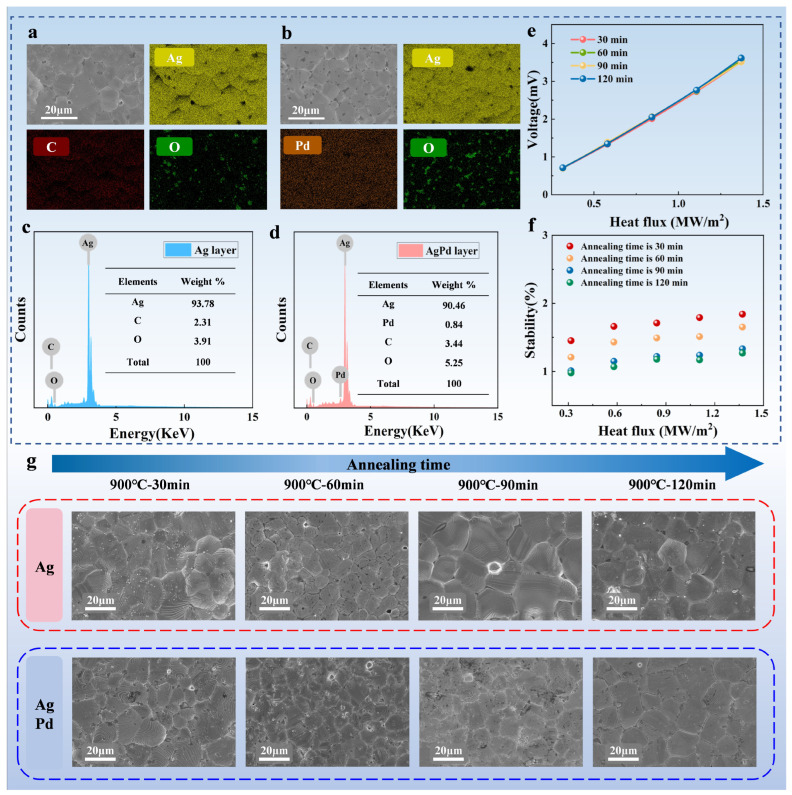
Microstructural characterization and annealing-time effect of the Ag–AgPd thick-film HFS. (**a**) SEM image and EDS elemental mapping of the Ag thick film. (**b**) SEM image and EDS elemental mapping of the AgPd thick film. (**c**) EDS spectrum of the Ag thick film. (**d**) EDS spectrum of the AgPd thick film. (**e**) Output voltage as a function of heat-flux density under different annealing times. (**f**) Annealing-time-dependent stability error under different heat-flux densities. (**g**) SEM images of the Ag and AgPd thick films annealed at 900 °C for 30, 60, 90, and 120 min.

Figure 2g shows the surface morphology of the Ag and AgPd thick films after annealing at 900 °C for different durations. For the Ag layer, the grain-like surface features become more distinguishable with increasing annealing time. For the AgPd layer, the film remains relatively continuous, and the morphology variation is less pronounced than that of the Ag layer. These observations indicate that annealing duration affects the surface morphology of the printed thick films.

Figure 2e shows the output voltage as a function of heat-flux density for samples annealed for 30, 60, 90, and 120 min. The output voltage increases approximately linearly with the applied heat-flux density for all annealing durations, indicating that the Ag–AgPd thick-film thermopile HFS maintains a near-linear heat-flux response after the different post-annealing treatments. Figure 2f compares the annealing-time-dependent stability error under different heat-flux densities. Longer annealing tended to reduce the stability error, whereas the calibration response remained comparable among the tested annealing durations. This result suggests that the annealing-induced morphology evolution mainly influenced the output stability, while the basic heat-flux calibration response remained nearly unchanged within the tested range. The 30 min sample already showed a near-linear calibration response and acceptable output stability under the tested heat-flux range. Therefore, considering the balance among calibration response, output stability, processing efficiency, and avoidance of unnecessary prolonged thermal exposure, annealing at 900 °C for 30 min was selected as the representative annealing condition for the subsequent calibration and dynamic-response tests.

The microstructure and elemental composition of the printed Ag and AgPd thick films were further characterized to verify the formation of the thermopile sensing layer. As shown in Figure 2a, the sintered Ag thick film exhibits a continuous surface morphology with distinguishable grain-like features. The corresponding EDS elemental mapping and spectrum in Figure 2a,c show that Ag is the dominant element in the observed region, while minor C and O signals are mainly associated with the thick-film preparation process and surface exposure. Figure 2b,d shows the SEM image, EDS elemental mapping, and elemental spectrum of the AgPd thick film after sintering. The AgPd layer also forms a continuous thick-film structure. Ag and Pd are distributed in the observed region, and the EDS spectrum confirms the coexistence of Ag and Pd in the printed AgPd layer. According to the reported Ag–Pd phase relationships, Ag–Pd alloying or ordered Ag–Pd phases may occur in Ag–Pd systems under thermal treatment conditions [22,23]. Therefore, the AgPd thick-film in this work is described as an Ag–Pd-containing thick-film layer based on the EDS-confirmed coexistence of Ag and Pd. These results indicate that the Ag and AgPd thick-film layers were successfully prepared by the screen-printing and sintering process, providing the material basis for the Ag–AgPd thermopile structure.

Since the sintering and post-annealing treatments were carried out in air, slight oxygen-related signals were observed in the EDS results. These trace oxygen signals may originate from surface adsorbates, oxygen-containing residues from the thick-film preparation process, or surface-related oxygen species. The EDS results indicate that the Ag thick film is mainly composed of Ag, whereas the AgPd thick film contains both Ag and Pd. Together with previous reports on the limited high-temperature stability of Ag oxide and PdO, these EDS observations suggest that pronounced oxidation was not evident in the Ag and AgPd thick films after the 900 °C air treatment [24,25]. The SEM images further show that the Ag and AgPd thick films maintained continuous surface morphologies after high-temperature treatment, without obvious peeling, cracking, or severe structural damage.

### 3.2. Heat-Flux Calibration and Performance Evaluation

The representative Ag–AgPd thick-film HFS annealed at 900 °C for 30 min was calibrated using the comparison calibration method described in Section 2.3. In this test, the fabricated HFS and the GD-C0-5M reference sensor were exposed to identical stepwise laser-heating conditions, and the steady-state HFS output voltage was correlated with the reference heat-flux density. The performance parameters, including sensitivity, linearity, nonlinearity, hysteresis, repeatability, sample-to-sample consistency, accuracy-test deviation, and repeated-loading stability, were calculated according to the definitions provided in the Supplementary Information. Sample-to-sample consistency was evaluated using nominally identical fabricated devices according to the definition provided in the Supplementary Information.

**Figure 3:**
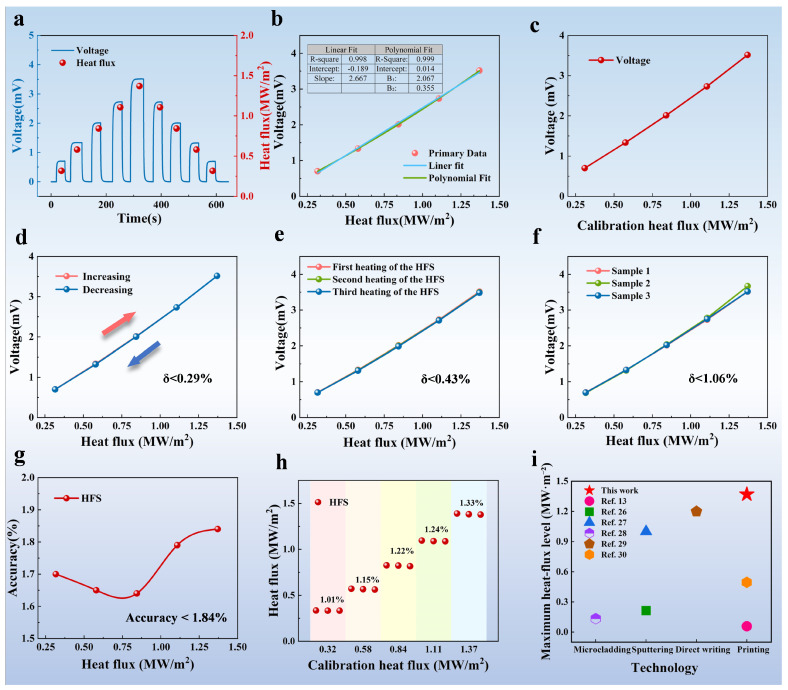
Static calibration and performance evaluation of the Ag–AgPd thick-film thermopile HFS. (**a**) Voltage response and reference heat flux during stepwise laser calibration. (**b**) Voltage–calibration heat-flux relationship with linear and second-order polynomial fitting results. (**c**) Output voltage as a function of calibration heat flux. (**d**) Hysteresis test during increasing and decreasing heat-flux loading. (**e**) Repeatability under three heating cycles. (**f**) Sample-to-sample consistency among three HFS samples. (**g**) Accuracy-test deviation under different calibration heat-flux levels. (**h**) Repeated heat-flux loading stability of the representative HFS. (**i**) Comparison of maximum heat-flux levels and fabrication technologies of representative thermopile-type heat-flux sensors.

The static calibration results under laser heating are shown in Figure 3. Figure 3a shows the laser-heating test process, in which the output voltage of the fabricated HFS was recorded under different heat-flux levels. As shown in Figure 3b, the reference heat-flux density increased from 0.32 to 1.37 MW/m^2^, and the output voltage of the fabricated HFS increased monotonically with the reference heat-flux density. The maximum output voltage was approximately 3.51 mV. Figure 3c shows the output voltage of the fabricated HFS as a function of the reference calibration heat flux.

The relationship between the output voltage $V_{\mathrm{out}}$ and the applied heat-flux density *q* was first fitted using a linear function:

(1) $$V_{\mathrm{out}}=B_{1}q+C$$

where $B_{1}$ is the heat-flux sensitivity and *C* is the intercept. The fitted sensitivity was 2.67 μV/(kW/m^2^), with an intercept of −0.19. The coefficient of determination was higher than 0.998. The nonlinearity, defined as the maximum deviation between the measured output and the linear fitting curve divided by the full-scale output, was 1.83%. These results show that the fabricated Ag–AgPd thick-film HFS provides a near-linear voltage response in the heat-flux range of 0.32–1.37 MW/m^2^.

The minimum detectable heat-flux density was further estimated from the baseline voltage fluctuation before heating. A 19 s pre-heating voltage record containing 759 data points was used to evaluate the baseline noise. The standard deviation of the baseline signal was $2.601\times 10^{-7}$ V, corresponding to 0.000260 mV. Using the calibrated sensitivity of 2.67 mV/(MW/m^2^), the minimum detectable heat-flux density was estimated according to $q_{\min}=3\sigma_{V}/S$, where $\sigma_{V}$ is the baseline voltage standard deviation and *S* is the calibrated sensitivity. The estimated $q_{\min}$ was 0.000292 MW/m^2^, namely 0.292 kW/m^2^.

To examine the nonlinear component of the calibration response, the calibration data were also fitted using a quadratic polynomial:

(2) $$V_{\mathrm{out}}=B_{2}q^{2}+B_{1}q+C.$$

The fitted coefficients were $B_{2}=0.36$, $B_{1}=2.07$, and C=0.01, with $R^{2}>0.999$. The quadratic fitting result further confirms that the output voltage follows the reference heat-flux density closely over the tested range, while the linear fitting relationship remains suitable for the primary calibration of the HFS.

The hysteresis, repeatability, sample-to-sample consistency, accuracy-test deviation, and repeated-loading stability results are summarized in Figure 3d–h. As shown in Figure 3d, the loading and unloading curves exhibit only a small difference, and the hysteresis error is below 0.29%. Figure 3e shows the repeatability results obtained from repeated calibration tests under the same conditions, with a repeatability error below 0.43%. Figure 3f shows the sample-to-sample consistency test, where the maximum consistency error is 1.06%. Figure 3g shows the accuracy-test deviation under different calibration heat-flux levels, and the maximum accuracy-test deviation was 1.84% over the tested heat-flux range. This accuracy-test deviation is different from the nonlinearity error of 1.83%, which was calculated from the maximum deviation between the measured output and the linear fitting curve. Figure 3h presents the repeated heat-flux loading stability of the representative HFS annealed at 900 °C for 30 min. The stability errors under the selected heat-flux levels were 1.01%, 1.15%, 1.22%, 1.24%, and 1.33%, respectively. The maximum stability error was 1.33%, indicating that the fabricated Ag–AgPd thick-film thermopile HFS maintained stable output behavior under the tested repeated-loading conditions. These calibration results indicate that the fabricated HFS can provide a repeatable voltage response to the reference heat-flux density over the tested MW/m^2^-level range.

**Figure 4:**
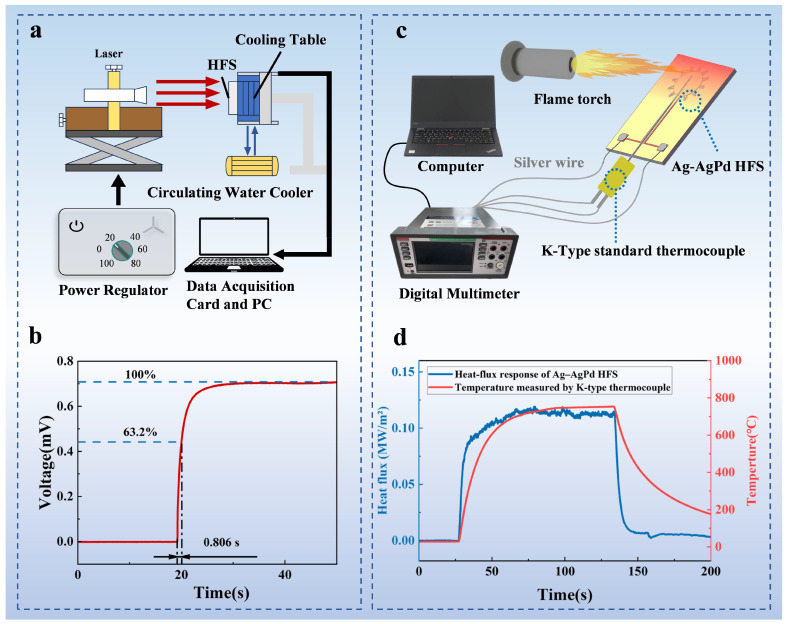
Dynamic-response and flame-heating evaluation of the Ag–AgPd thick-film HFS. (**a**) Schematic of the laser step-heating test. (**b**) Transient voltage response under laser step excitation. (**c**) Schematic of the flame-heating test. (**d**) Temperature and baseline-corrected equivalent heat-flux responses during heating and cooling.

The transient response of the fabricated HFS was evaluated by laser step-heating, as shown in Figure 4a. During the test, a sudden laser heat input was applied to the sensing region, and the output voltage was recorded during the laser-on process. As shown in Figure 4b, the transient voltage response follows a first-order-like trend after the step heat-flux excitation. The time constant is a key parameter for describing the dynamic behavior of this response. Based on the 63.2% steady-state definition, the time constant was calculated to be 0.806 s. The steady-state output voltage under the step-heating condition was approximately 0.704 mV. These results indicate that the fabricated HFS can capture transient heat-flux variations under the present laser step-heating condition.

To further evaluate the high-temperature output behavior of the fabricated HFS under practical heating conditions, a flame-heating test was conducted, as shown in Figure 4c. During the test, a flame torch was used to directly heat the sensing region of the sensor. The heating process was maintained for approximately 120 s, while a K-type thermocouple was used to monitor the local temperature near the sensing region, and the output voltage of the HFS was recorded simultaneously.

Figure 4d presents the temperature and baseline-corrected equivalent heat-flux responses during the heating and cooling processes. The equivalent heat-flux response was converted from the measured voltage using the laser-calibration sensitivity according to $q_{\mathrm{eq}}\left(t\right)=\left[V\left(t\right)-V_{0}\right]/2.67$, where V(t) is the measured voltage in mV, $V_{0}$ is the average baseline voltage before flame heating, and 2.67 mV/(MW/m^2^) is the laser-calibration sensitivity. This conversion was used to describe the relative heat-flux response under flame heating rather than to provide an independent flame heat-flux calibration. After the flame was applied, the equivalent heat-flux response increased together with the monitored temperature. The local temperature reached about 800 °C, and the equivalent heat-flux response remained nearly constant with only slight fluctuations during the sustained heating stage. After the flame was removed, both the temperature and the equivalent heat-flux response decreased with the cooling process. The consistent variation trend between the equivalent heat-flux response and the monitored temperature further confirms the thermoelectric response of the Ag–AgPd junction under the tested flame-heating condition.

### 3.3. Comparison with Reported Thermopile-Type Heat-Flux Sensors

To place the present Ag–AgPd thick-film thermopile HFS in the context of reported thermopile-type HFSs, Figure 3i compares the maximum reported heat-flux levels and fabrication routes of selected devices from Refs. [13,26,27,28,29,30]. Under the tested calibration conditions, the present Ag–AgPd thick-film thermopile HFS was calibrated up to 1.37 MW/m^2^ using screen printing and multi-step sintering on a SUS430 stainless-steel substrate.

The comparison in Figure 3i provides a graphical overview of reported maximum heat-flux levels and fabrication routes. Because the compared devices differ in structure, substrate, operating condition, and calibration method, the comparison is used as a contextual reference. The present device demonstrates an MW/m^2^-level calibrated heat-flux response over 0.32–1.37 MW/m^2^, indicating the feasibility of the Ag–AgPd thick-film thermopile HFS for high-temperature heat-flux measurement.

## 4. Conclusions

In summary, an Ag–AgPd thick-film thermopile HFS was fabricated on a SUS430 stainless-steel substrate by screen printing and multi-step sintering. The sensor consisted of an insulating layer, an Ag–AgPd thermopile sensing layer, and a thermal-resistance layer.

Microstructural characterization confirmed the formation of continuous Ag and AgPd thick-film layers. Considering output response, stability, processing efficiency, and avoidance of unnecessary prolonged thermal exposure, the sample post-annealed at 900 °C for 30 min was selected as the representative condition for subsequent tests.

Laser comparison calibration showed a near-linear output response over 0.32–1.37 MW/m^2^, with $R^{2}>0.998$, a sensitivity of 2.67 μV/(kW/m^2^), a nonlinearity of 1.83%, a hysteresis error below 0.29%, a repeatability error below 0.43%, a sample-to-sample consistency error of 1.06%, and a maximum repeated-loading stability error of 1.33%. The estimated minimum detectable heat-flux density was 0.292 kW/m^2^.

The laser step-heating test yielded a time constant of 0.806 s, and the flame-heating test showed that the baseline-corrected equivalent heat-flux response remained stable during approximately 120 s of exposure at about 800 °C. These results indicate that the Ag–AgPd thick-film thermopile HFS is a feasible sensing approach for MW/m^2^-level heat-flux measurement on high-temperature metallic surfaces. The proposed screen-printed thick-film structure also provides a practical basis for further development of integrated heat-flux sensors for high-temperature metallic-component monitoring.

## Data Availability

Data will be made available on request.

## Supplementary Materials

The following supporting information can be downloaded at: https://www.mdpi.com/article/10.3390/s26134030/s1, Figure S1: Dimensional parameters of the Ag–AgPd heat-flux sensor; Figure S2: Supplementary structural design and physical photograph of the Ag–AgPd thick-film thermopile HFS; Figure S3: Microstructural characterization of the thermal-resistance layer after sintering; Figure S4: Laser calibration platform for heat-flux testing.

## References

[1] Han J.-C., Dutta S., Ekkad S. (2013). Gas Turbine Heat Transfer and Cooling Technology.

[2] Dunn M.G. (2001). Convective heat transfer and aerodynamics in axial flow turbines. J. Turbomach..

[3] Jackowski T., Elfner M., Bauer H.-J. (2021). Experimental study of impingement effusion-cooled double-wall combustor liners: Thermal analysis. Energies.

[4] Mazzei L., Andreini A., Facchini B., Bellocci L. A 3D coupled approach for the thermal design of aero-engine combustor liners. Proceedings of the ASME Turbo Expo: Turbomachinery Technical Conference and Exposition.

[5] Nicholas T.E.W., Pernak M.J., Scobie J.A., Lock G.D., Tang H. (2023). Transient heat transfer and temperatures in closed compressor rotors. Appl. Therm. Eng..

[6] Li Z., Yin J., Wang G., Liang H., Zhang C., Huang M., Liu Y., Zhang J. (2022). Dynamic calibration of a thin-film heat-flux sensor in time and frequency domains. Sensors.

[7] Morelli U.E., Barral P., Quintela P., Rozza G., Stabile G. (2023). Novel methodologies for solving the inverse unsteady heat transfer problem of estimating the boundary heat flux in continuous casting molds. Int. J. Numer. Methods Eng..

[8] Perakis N., Haidn O.J. (2019). Inverse heat transfer method applied to capacitively cooled rocket thrust chambers. Int. J. Heat Mass Transf..

[9] Dickhoff J., Kusterer K., Horikawa A., Bohn D. (2020). Development of an air cooled borescope for infrared thermal load monitoring in industrial gas turbine combustors and operational experience. Int. J. Gas Turbine Propuls. Power Syst..

[10] Manara J., Zipf M., Stark T., Arduini M., Ebert H.P., Tutschke A., Hallam A., Hanspal J., Langley M., Hodge D. (2017). Long wavelength infrared radiation thermometry for non-contact temperature measurements in gas turbines. Infrared Phys. Technol..

[11] Wang Y., Zhang C., Yang S., Li Y., Zheng R., Cai H., Zhang Y., Zhao N., Kang Z., Guo Y. (2023). In situ integration of high-temperature thin-film sensor for precise measurement of heat flux and temperature on superalloy substrate. IEEE Sens. J..

[12] Chen X., Tao B., Zhao R., Yang K., Li Z., Xie T., Xia Y. (2024). Fast-response thin film heat flux sensors for harsh environments. IEEE Sens. J..

[13] Zhang T., Tan Q., Lyu W., Lu X., Xiong J. (2019). Design and fabrication of a thick film heat flux sensor for ultra-high temperature environment. IEEE Access.

[14] Siroka S., Berdanier R.A., Thole K.A., Chana K., Haldeman C.W., Anthony R.J. (2020). Comparison of thin film heat flux gauge technologies emphasizing continuous-duration operation. J. Turbomach..

[15] Wen M., Guan X., Li H., Ou J. (2020). Temperature characteristics of thick film resistors and its application as a strain sensor with low temperature-sensitivity. Sens. Actuators A Phys..

[16] Wang J., Tian W., Wang Y., Zhou H., He Y., Wang Y., Li T. (2022). Micromachined thermocouple for rapid detection of ultrahigh heat flux at high temperature. IEEE Trans. Ind. Electron..

[17] Riches S.T., Whitmarsh J., Hamilton D. Silver thick film based insulated metal substrates for high temperature power applications. Proceedings of International Conference and Exhibition on High Temperature Electronics Network (HiTEN).

[18] Zhu K., Tang L., Qian X., Ni H., Chen M., Wu C. (2025). A novel AgPd-Pt thick-film strain gauge with excellent long-term stability and reliable strain sensing from room temperature to 1000 °C. Measurement.

[19] Balakrishnan T.S., Sultan M.T.H., Shahar F.S., Saravanakumar Y.N., Chandran N.K., Sultan M.T.H., Uthayakumar M., Korniejenko K., Mashinini P.M., Najeeb M.I., Krishnamoorthy R.R. (2025). Aerospace steel: Properties, processing, and applications. Aerospace Materials: Novel Technologies and Practical Applications.

[20] Zarr R.R. (1994). Control stability of a heat-flow-meter apparatus. J. Therm. Insul. Build. Envel..

[21] Bordia R.K., Kang S.-J.L., Olevsky E.A. (2017). Current understanding and future research directions at the onset of the next century of sintering science and technology. J. Am. Ceram. Soc..

[22] Karakaya I., Thompson W.T. (1988). The Ag–Pd (Silver–Palladium) system. Bull. Alloy Phase Diagr..

[23] Kitchin J.R., Reuter K., Scheffler M. (2008). Alloy surface segregation in reactive environments: A first-principles atomistic thermodynamics study of Ag_3_Pd(111) in oxygen atmospheres. Phys. Rev. B.

[24] Garner W.E., Reeves L.W. (1954). The thermal decomposition of silver oxide. Trans. Faraday Soc..

[25] Badica P., Lőrinczi A. (2024). A review on preparation of palladium oxide films. Coatings.

[26] Li X., Cui Z., Sun D., Chen Q., He G., Liu B., Hai Z., Chen G., Jia Z., Yao Z. (2022). Development of thin film heat flux sensor based on transparent conductive oxide thermopile with antireflective coating. Sens. Rev..

[27] Fu X., Lin Q., Peng Y., Liu J., Yang X., Zhu B., Ouyang J., Zhang Y., Xu L., Chen S. (2020). High-temperature heat flux sensor based on tungsten–rhenium thin-film thermocouple. IEEE Sens. J..

[28] Wang Y., Ling Y., Cao J., Wu L., Meng L., Zhang S., Li N., Bai W., Ouyang T., Zeng X. (2023). Conformal fabrication of high-sensitivity embedded thick film thermopile heat flux sensors: Design, preparation, and performance. IEEE Sens. J..

[29] Xu L., Zhou X., Huang Y., Wang Y., Shao C., Li Y., Wang L., Yang Q., Sun D., Chen Q. (2024). Design and fabrication of metal spherical conformal thin film multisensor for high-temperature environment. Chin. J. Aeronaut..

[30] Dong H., Lu M., Wang W., Tan Q. (2024). High temperature heat flux sensor with ITO/In_2_O_3_ thermopile for extreme environment sensing. Microsyst. Nanoeng..

