# Supplementary material for: Fabrication and Characterization of an Ag–AgPd Thick-Film Thermopile Heat-Flux Sensor for High-Temperature Applications
Source: Sensors (Basel). 2026 Jun 25;26(13):4030. doi: 10.3390/s26134030 (PMC13364419; doi:10.3390/s26134030)
Supplement: Supplementary file 1 [file sensors-26-04030-s001.zip › sensors-4374506-supplementary.pdf]

Supplementary Information

Fabrication and Characterization of an Ag–AgPd Thick-Film Thermopile

Heat-Flux Sensor for High-Temperature Applications

Zhichun Liu, Fei Chen, Zhixuan Su, Heng Wang, Jinghan Si,
Junyang Chen, Zihan Du, and Zhenyin Hai

School of Aerospace Engineering, Xiamen University, Xiamen 361005, China

Corresponding author: Zhenyin Hai (haizhenyin@xmu.edu.cn)

S1. Working Principle

The operating principle of the HFS can be described on the basis of the first law of thermodynamics, Fourier's one-dimensional heat-conduction law, and the Seebeck effect. Under one-dimensional steady-state heat conduction, the temperature distribution in the thermal-resistance layer satisfies

$$\frac{d^2T}{dx^2} = 0. \tag{S1}$$

When heat flux passes through the thermal-resistance layer in the thickness direction, a stable temperature difference is established between its upper and lower surfaces. According to Fourier's law, the heat-flux density q can be expressed as

$$q = -\lambda \frac{dT}{dx}, \tag{S2}$$

where λ is the thermal conductivity of the thermal-resistance layer. Combining the governing equation with the boundary conditions gives

$$q = -\lambda \frac{T_1 - T_2}{\delta} = \lambda \frac{T_2 - T_1}{\delta}, \tag{S3}$$

where T_2 and T_1 denote the temperatures at the hot and cold sides, respectively, and δ is the thickness of the thermal-resistance layer.

The thermopile serves as the sensing element. According to the Seebeck effect [1], when two dissimilar conductors are subjected to a temperature difference, a thermoelectric voltage is generated:

$$V_{\text{out}} = nS_{AB}\Delta T, \tag{S4}$$

where n is the number of thermocouple pairs, ΔT is the temperature difference between the hot and cold junctions, and S_{AB} is the Seebeck coefficient of the Ag–AgPd thermocouple pair. Combining (S3) and (S4), the heat-flux sensitivity can be written as

$$\frac{V_{\text{out}}}{q} = \frac{nS_{AB}\delta}{\lambda}. \tag{S5}$$

This relationship provides the theoretical foundation for the calibration and performance analysis presented in the main text.

S2. Performance Parameter Evaluation

To quantitatively evaluate the fabricated HFS, the principal performance parameters considered in this study include stability, hysteresis, repeatability, accuracy, and response time.

Stability (or drift) characterizes the ability of the HFS to maintain a steady output under a constant heat-flux input:

$$e_{\text{s}} = \frac{\Delta U_{\text{max}}}{U_{\text{FS}}} \times 100\%, \tag{S6}$$

where e_{s} is the stability error, ΔU_{max} is the maximum output-voltage variation during the holding period, and U_{FS} is the full-scale output voltage.

Hysteresis characterizes the difference between the sensor outputs obtained during increasing and decreasing heat-flux loading:

$$e_{\text{h}} = \frac{\Delta H_{\text{max}}}{U_{\text{FS}}} \times 100\%, \tag{S7}$$

where ΔH_{max} is the maximum deviation between the loading and unloading outputs.

Repeatability describes the consistency of repeated measurements performed under identical conditions:

$$e_{\text{r}} = \frac{\Delta R_{\text{max}}}{U_{\text{FS}}} \times 100\%, \tag{S8}$$

where ΔR_{max} is the maximum deviation among repeated calibration results.

Accuracy characterizes the deviation between the heat-flux density measured by the tested HFS and the reference value provided by the standard sensor:

$$e_{\text{a}} = \frac{|q_{\text{m}} - q_{\text{ref}}|}{q_{\text{FS}}} \times 100\%, \tag{S9}$$

where q_{m} is the heat-flux density measured by the tested HFS, q_{ref} is the reference heat-flux density, and q_{FS} is the full-scale heat-flux density in the calibration range.

The response time τ is defined as

$$\tau = t_{0.632}, \tag{S10}$$

where $t_{0.632}$ is the time required for the sensor output to reach 63.2% of its steady-state value after a step thermal excitation.

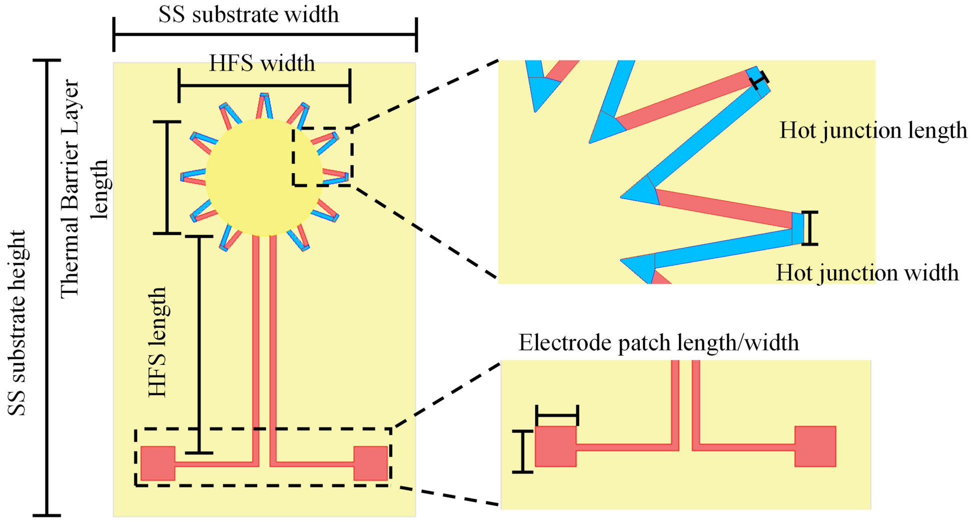

Fig. S1: Dimensional parameters of the Ag–AgPd heat-flux sensor.

S3. Geometrical Parameters

Fig. S1 summarizes the geometric dimensions of the fabricated Ag–AgPd thick-film HFS. The sensing-pattern area was approximately 12 mm × 10 mm, with an overall printed-layer thickness of about 0.03 mm. The SUS430 stainless-steel substrate measured 30 mm × 20 mm × 1 mm, and the hot-junction region was approximately 0.2 mm × 2 mm. The Al_2O_3 solder-pad support had nominal dimensions of 22 mm × 28 mm × 1 mm, with a local solder-pad section of 22 mm × 10 mm × 1 mm. The welding pads were approximately 3 mm × 3 mm × 1 mm and were connected to 0.3-mm-diameter Ag wires.

S4. Supplementary Structural Information

Fig. S2 provides supplementary structural information for the Ag–AgPd thick-film thermopile HFS. The three-dimensional schematic in Fig. S2(a) shows the overall multilayer sensor structure, lead connection region, and sensing region. Fig. S2(b) presents an enlarged view of the Ag–AgPd thermopile sensing region, showing the interlaced Ag and AgPd thermoelectric legs around the thermal-resistance layer. Fig. S2(c) shows a photograph of the fabricated HFS with the sensing pattern and electrical lead connections.

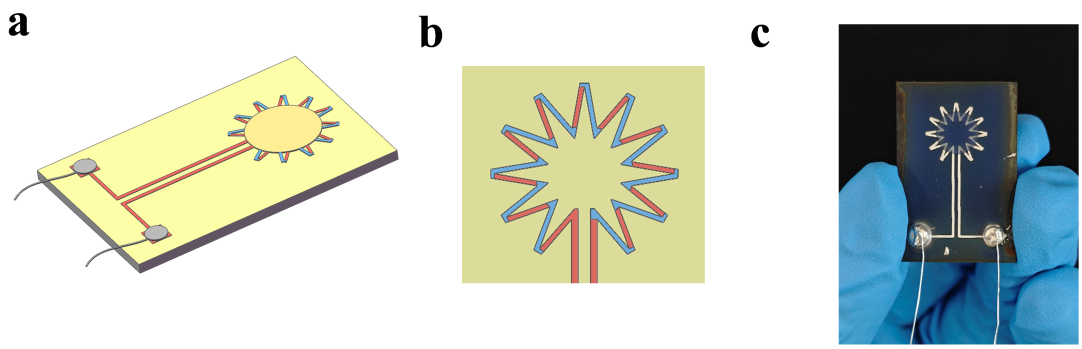

Fig. S2: Supplementary structural design and physical photograph of the Ag–AgPd thick-film thermopile HFS: (a) three-dimensional schematic of the sensor structure; (b) enlarged schematic of the Ag–AgPd thermopile sensing region; and (c) photograph of the fabricated HFS.

Fig. S3 presents the microstructural characterization and elemental analysis of the thermal-resistance layer after sintering. The SEM image shows that the surface of the thermal-resistance layer exhibits a continuous interwoven flake-like morphology with randomly wrinkled features and pronounced micro-topographical undulations, together with a small number of minute particulate impurities or pores. This wrinkled microstructure helps form a continuous structural

layer over the sensing region. In addition, the thermal-resistance layer provides through-thickness thermal resistance for establishing a temperature difference during heat-flux loading. The corresponding EDS surface elemental distribution of Ca, Al, Si, C, and O shows that the elemental composition is consistent with the expected stoichiometric ratio, indicating the formation of a compositionally uniform surface film.

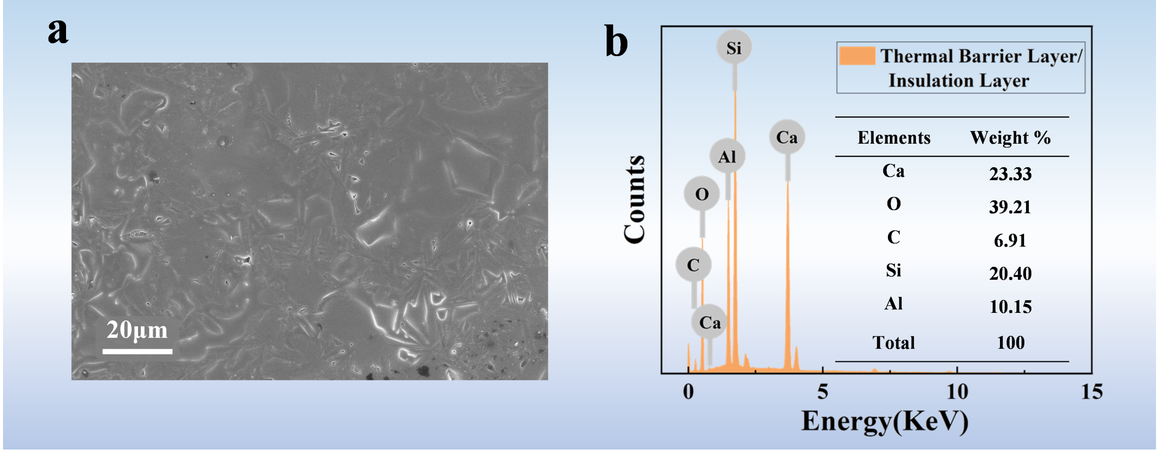

Fig. S3: Microstructural characterization of the thermal-resistance layer after sintering: (a) SEM image and (b) corresponding elemental analysis results.

S5. Testing Equipment

An OTF-1200X tube furnace (Hefei Kejing Material Technology Co., Ltd., China) was used for sintering and thermal testing of the thick-film HFS. A DAQ6510 digital multimeter/data-acquisition unit (Tektronix, USA) was used to record the thermoelectric output of the HFS. A 1064 nm fiber laser (YLM-QCW, IPG Photonics, USA) with a maximum power of 300 W was used as the controllable heat source for heat-flux calibration and laser step-heating tests. A calibrated reference heat-flux sensor (GD-C0-5M, Xi'an Kretek Science and Trade Co., Ltd., China) was used to determine the applied heat-flux density under different laser powers during comparison calibration.

Scanning electron microscopy (SUPRA55 SAPPHIRE, Carl Zeiss AG, Germany) was used for microstructural characterization, and energy-dispersive spectroscopy on the same platform was used to analyze the elemental distribution of the Ag–AgPd thick films. Fig. S4 shows the laser calibration platform used for heat-flux testing of the fabricated sensors.

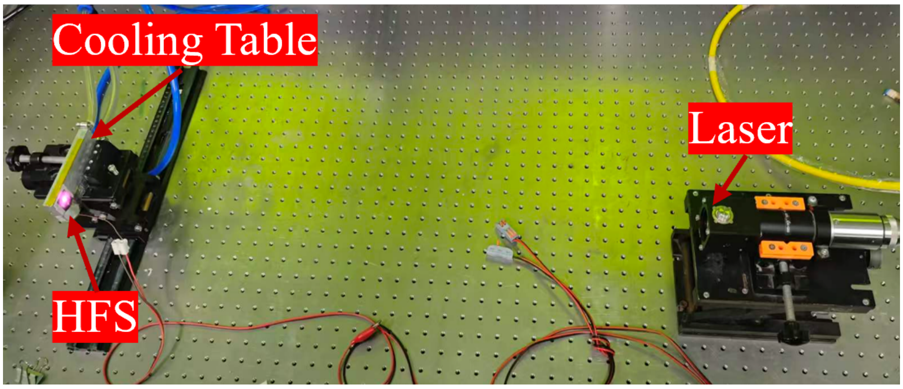

Fig. S4: Laser calibration platform for heat-flux testing.

References

[1] Wang, J.; Tian, W.; Wang, Y.; Zhou, H.; He, Y.; Wang, Y.; Li, T. Micromachined thermocouple for rapid detection of ultrahigh heat flux at high temperature. *IEEE Trans. Ind. Electron.* **2022**, *69*, 2099–2106.
